# Integrating subjective perceptions and objective video analysis to identify challenges in laparoscopic suturing: a cross-sectional study to enhance surgical training

**DOI:** 10.1038/s41598-026-39914-5

**Published:** 2026-02-14

**Authors:** Chidozie Ogbonnaya, Shizhou Li, Changshi Tang, Baobing Zhang, Paul Sullivan, Mustafa Suphi Erden, Benjie Tang

**Affiliations:** 1 Surgical Skills Centre, Respiratory Medicine and Gastroenterology, School of Medicine, Ninewells Hospital and Medical School, Dundee Institute for Healthcare Simulation, University of Dundee, Dundee, DD1 9SY UK; 2 Hammersmith Hospital, Imperial College, Hammersmith Campus, London, W12 0HS UK; 3 School of Medicine, University of Edinburgh, Edinburgh, EH8 9YL UK; 4 School of Engineering and Physical Sciences, Heriot Watt University, Edinburgh, EH14 4AS UK

**Keywords:** Laparoscopic suturing, Surgical training, Skill acquisition, Knot tying, Simulation, Objective performance assessment., Health care, Medical research

## Abstract

Laparoscopic suturing remains one of the most technically demanding skills in minimally invasive surgery. This study aimed to identify the key technical and cognitive challenges encountered during laparoscopic suturing through both subjective perceptions and objective performance analysis. It was also sought to inform the development of more effective, targeted training strategies to enhance laparoscopic suturing training proficiency. A cross-sectional study was conducted with 33 laparoscopic surgeons, 22 novices and 11 experts. A Delphi consensus among six expert surgeons identified four core subtasks which formed the basis of a structured survey. Participants performed standardized laparoscopic suturing on animal tissue using a box trainer before completing the questionnaire. Objective assessments using the Global Operative Assessment of Laparoscopic Skills (GOALS) evaluated time to completion, needle handling, knot tying quality, tissue manipulation, and tension maintenance through video analysis. Knot tying was reported as the most challenging task by 42.4% of participants, followed by needle handling at 27.3% and maintaining suture tension at 21.2%. No significant difference in perceived difficulty was observed between novice and expert surgeons. Objective GOALS-based analysis demonstrated that expert surgeons significantly outperformed novices across all metrics. Mean time to complete suturing was 5.7 ± 0.8 min for experts compared with 8.4 ± 1.2 min for novices (*P* < 0.001). Needle handling scores were 4.5 ± 0.3 versus 2.9 ± 0.5 (*P* < 0.001). Knot tying quality was 4.6 ± 0.4 versus 2.8 ± 0.6 (*P* < 0.001). Tissue manipulation scores were 4.4 ± 0.3 versus 3.0 ± 0.5 (*P* < 0.001). Tension maintenance scores were 4.5 ± 0.4 versus 2.7 ± 0.6 (*P* < 0.001). This study demonstrates that technical challenges in laparoscopic suturing persist across all experience levels. Integrating subjective perceptions with objective GOALS-based video analysis provides a comprehensive assessment of performance differences. Targeted simulation training focusing on knot tying, needle manipulation, hand positioning, and motion efficiency is essential to enhance suturing proficiency.

## Introduction

Minimally invasive surgery (MIS) has revolutionized the field of surgery by offering patients numerous benefits, such as faster recovery times, less postoperative pain, and shorter hospital stays^1–3^. Despite these advantages, there remain significant challenges in the field, particularly with laparoscopic suturing^4,5^. Laparoscopic suturing is often cited as one of the most technically demanding skills for surgeons, largely due to the unique constraints of the laparoscopic environment^6,7^. Unlike open surgery, where surgeons can directly handle instruments and sutures, laparoscopic surgery is performed through small incisions, using long, rigid instruments controlled from fixed entry points^8^. This setup limits the range of motion, complicates depth perception, and removes the tactile feedback surgeons rely on during open procedures^6^. These challenges make key tasks such as needle handling, tissue manipulation, knot tying, and maintaining consistent suture tension much more difficult to execute. Mastering these essential skills is crucial for successful surgical outcomes, reducing the risk of intraoperative complications and ensuring the safety and well-being of patients^4,9^. Improving laparoscopic suturing skills is critical for advancing the field of MIS and enhancing patient care. Simulation, including virtual reality and tactile feedback, has shown promise in offering trainees the opportunity to practice skills in a controlled, low-risk environment^10–12^.

Studies have demonstrated that difficulties in laparoscopic suturing persist across varying levels of experience, highlighting that basic laparoscopic skills do not necessarily correlate with proficiency in suturing techniques^6^. In particular, the manual dexterity required for intracorporeal knot-tying and suturing underscores the complexity of these tasks^13,14^. Given these ongoing challenges, this study focuses on identifying the specific difficulties surgeons face during laparoscopic suturing and exploring how tailored training approaches can enhance proficiency^15^. Knot tying, for example, is a crucial skill in many laparoscopic procedures, such as closing incisions or securing anastomoses. Difficulty in performing this task can result in increased operative time, poor surgical outcomes, and complications such as tissue damage or infection^16,17^.

Laparoscopic suturing remains one of the most technically demanding skills in minimally invasive surgery^18,19^. Despite its importance, there is currently no clear evidence regarding whether experienced surgeons share the same perspectives on the challenges of this skill. To address this gap, this study gathered insights from both novice and expert surgeons to identify the most technically demanding aspects of suturing, including needle handling, knot tying, tissue manipulation, and maintaining suture tension, using both subjective perceptions and objective performance analysis. The study also aimed to explore why these difficulties persist across different levels of expertise and to determine whether they differ between novice and expert surgeons. By understanding the underlying factors that contribute to these challenges, the findings are intended to inform the development of more effective and targeted training strategies, including simulation-based programs, to enhance laparoscopic suturing proficiency across all experience levels.

## Methods

This study employs a cross-sectional survey design to assess the perceived challenges in laparoscopic suturing among surgeons with varying levels of experience. The study was conducted in accordance with the Declaration of Helsinki and approved by the Institutional Review Board of University of Dundee (UOD-SMED-SLS-Staff-2024-24-117), and the research was conducted at the Surgical Skills Centre at Ninewells Hospital.

Participants were enrolled through voluntary recruitment at the Surgical Skills Centre during scheduled laparoscopic training workshops. Invitations were distributed via departmental email lists, and interested surgeons registered through an online form before participation. This ensured that all participants were practicing laparoscopic surgeons who met the inclusion criteria. Participants were categorized as novice or expert based primarily on the number of laparoscopic suturing procedures performed, with novices defined as surgeons who had performed fewer than 50 procedures and experts as those who had performed more than 200 procedures. Years of surgical experience were also recorded to provide context regarding overall surgical practice.

A Delphi consensus process was conducted with six expert laparoscopic surgeons; each having performed over 200 laparoscopic procedures requiring suturing. Through this process, a list of subtasks involved in laparoscopic suturing was identified and summarized in.

Table 1.

**Table 1:** Core laparoscopic suturing skills identified by the Delphi consensus agreement of six expert laparoscopic surgeons.

Needle manipulation	Knot tying	Instruments and tissue handling
Introduction of a needle into trainer box	Preparing and placing suture in right position	Keeping needle in laparoscopic view
Removal of a needle from trainer box	Formation of a C-loop for tying the first half of knot	Trajectory of instruments (time and motion)
Needle pick-up	Formation of reverse C-loop to tie the second half of knot	Correct force of tissue handling
Loading a needle in a holder with good position and direction	Formation of a square and secure knot	Flow of procedure and forward planning
Precise insertion of a needle through the tissue	Maintaining tension of the knot	Coordination and control of both instruments

These subtasks informed the design of a questionnaire for the survey. The questionnaire employed a Likert scale format, allowing participants to rate the difficulty level of each subtask rather than rank-ordering them. This approach enabled a more detailed analysis of perceived challenges. The cross-sectional survey aimed to explore the challenges surgeons face in laparoscopic suturing across different experience levels. Before completing the questionnaire, all participants provided informed consent, ensuring compliance with ethical research guidelines and maintaining participant confidentiality.

**Fig. 1:**
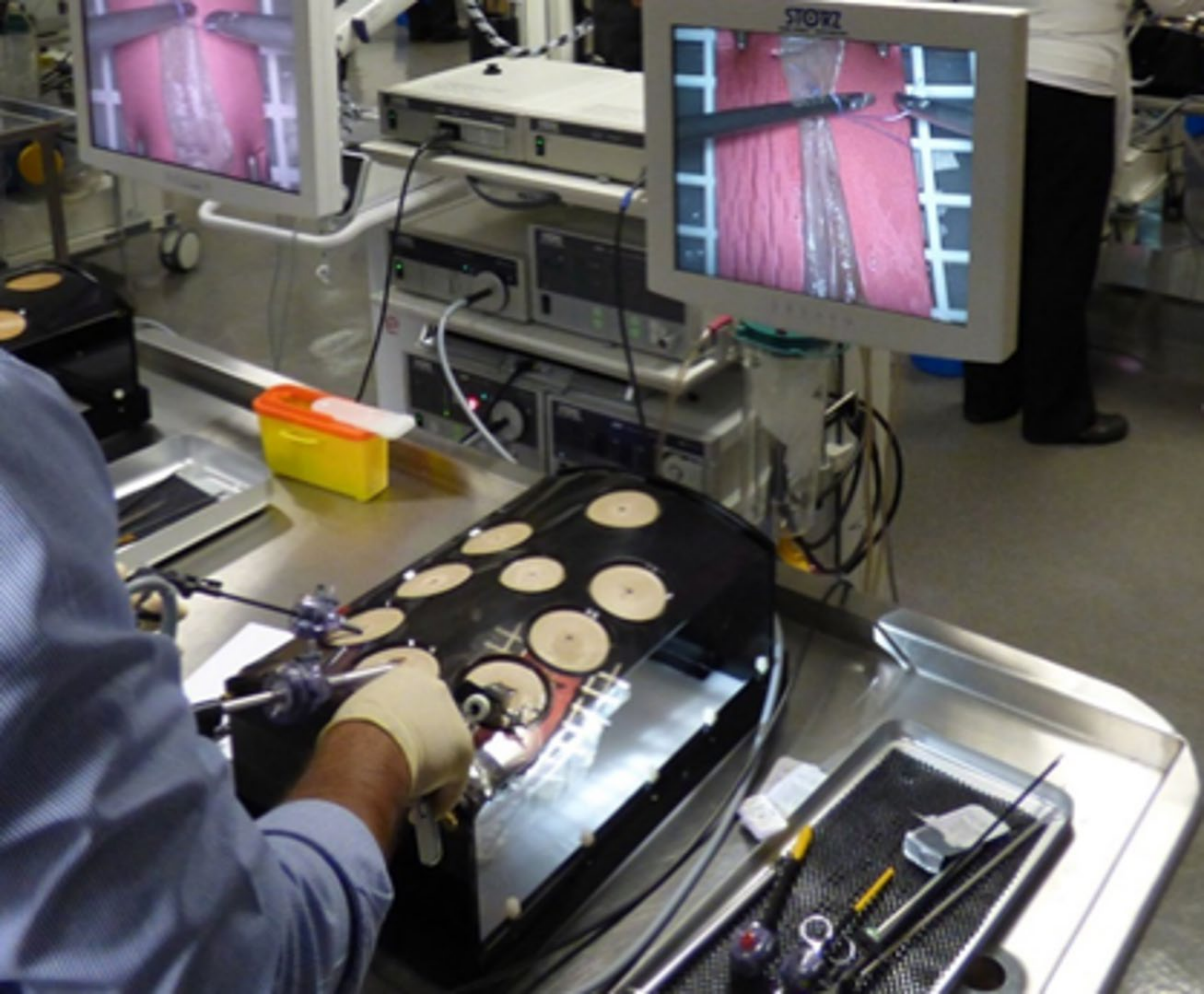
Laparoscopic surgical training box setup featuring camera system, surgical instruments, and simulated practice model for suturing and precision trai ning.

Prior to completing the questionnaire, both novice and expert surgeons performed laparoscopic suturing on animal tissue using a laparoscopic box and laparoscopic instruments over a standardized 3-centimeter incision, as shown in Fig. 1. The animal tissue utilized was a piece of cadaveric porcine small bowel. The cadaveric porcine small bowel was procured from a licensed company based in the United Kingdom (MEDMEAT LTD, Reg. No. 14558852, Park House, 200 Drake Street, Rochdale, United Kingdom, OL16 1PJ). Hand movements, wrist rotation, and needle positioning during suturing were recorded via video for subsequent analysis. There were no live animals used in this study, and therefore no animal ethics approval was required.

Standard laparoscopic instruments and a laparoscopic stack with a 2-dimensional monitor were used to perform the simulated laparoscopic suturing. Needle holders were introduced into the standard trainer box with a 60° manipulation angle, an elevation angle of 45° to 60°, and equal azimuth angles. Performance of hand movements, wrist rotation, and needle positioning were recorded during laparoscopic suturing to objectively assess motion quality, economy of movement, and ergonomic efficiency. This hands-on practice allowed participants to engage with the suturing tasks directly, providing them with an opportunity to experience the challenges involved before reflecting on their perceived difficulties. Additionally, it ensured that both novice surgical trainees and expert surgeons had immediate exposure to a simulation-based training setup before completing the survey.

The objective clinical evaluation of laparoscopic suturing skills was conducted for each participant using the Global Operative Assessment of Laparoscopic Skills (GOALS) scoring system^20^. Each suturing session was video recorded and later reviewed using the GOALS criteria to ensure standardized, objective scoring. Video assessments were performed independently by two experienced laparoscopic surgeons, each with over five years of operative and teaching experience in minimally invasive surgery. Both reviewers were trained in GOALS scoring procedures, and discrepancies in ratings were resolved through consensus to ensure inter-rater reliability. This approach allowed for direct comparison between participants’ subjective perceptions of difficulty and their observable performance outcomes. Knot quality was rated on a 1–5 GOALS scale based on symmetry, slippage, and tension. This evaluation focused on key performance metrics, including needle pass accuracy, knot-tying quality, time to completion, and error rate. Time to completion was measured in minutes from the first needle insertion to final knot securement using video timestamps. Needle pass accuracy and error rate were determined through detailed video review, with each missed target, tissue trauma, or improper entry angle recorded as an error. Suture tension consistency was evaluated visually based on the GOALS “tissue handling” domain, rating the surgeon’s ability to maintain even, appropriate tension throughout the task.

The study population consisted of 33 laparoscopic surgeons, including 22 novice surgeons and 11 expert surgeons, who all regularly performed laparoscopic procedures in specialties such as general surgery, gynaecology, and urology. Novice surgeons were defined as those with less than 10 years of surgical experience, while expert surgeons had more than 10 years of experience and had performed more than 200 laparoscopic procedures that required laparoscopic suturing. Surgeons who did not routinely perform laparoscopic suturing were excluded from the study to ensure the relevance of the responses.

Statistical analysis was performed using SPSS (Statistical Package for the Social Sciences), incorporating descriptive and exploratory inferential methods to examine trends in perceived difficulty levels. Frequencies and percentages were calculated to summarise participant characteristics and the proportion of novice and expert surgeons identifying each laparoscopic suturing task as challenging.

Categorical variables relating to perceived task difficulty were analysed using chi-square tests for independence to examine associations between surgeon experience level and perceived difficulty. Given the sample size and the presence of low expected frequencies in some response categories, these analyses were interpreted cautiously and are presented primarily to illustrate trends rather than provide definitive inferential conclusions.

Objective performance outcomes, including time to complete suturing and GOALS domain scores, were compared between novice and expert surgeons using independent-samples t-tests, as these outcome measures are continuous and appropriate for between-group comparisons.

No formal correction for multiple comparisons was applied, as the analyses were exploratory and intended to identify patterns and potential differences rather than establish confirmatory statistical inference. Findings are therefore interpreted in the context of effect magnitude and clinical relevance.

Graphical representations, including bar charts and stacked column charts, were used to visually compare subjective responses and objective performance outcomes between groups.

## Results

A total of 33 surgeons participated in the study, consisting of 22 novice surgeons and 11 expert surgeons.

Table 2 summarises the demographic and surgical experience of study participants. Expert surgeons had substantially more years of overall surgical practice (15.6 ± 3.4 vs. 5.8 ± 2.1) and laparoscopic experience (13.2 ± 3.1 vs. 4.6 ± 1.9) compared with novice surgeons. Experts also performed more laparoscopic procedures (420 ± 110 vs. 85 ± 40) and laparoscopic suturing cases (260 ± 75 vs. 35 ± 20). The distribution of surgical specialties: general surgery, gynaecology, and urology was similar between groups, demonstrating comparable representation across specialties.

**Table 2:** Demographic and experience characteristics of study Participants.

Variable	Novice surgeons (*n* = 22)	Expert surgeons (*n* = 11)
Mean years in surgical practice (± SD)	5.8 ± 2.1	15.6 ± 3.4
Mean years performing laparoscopic surgery (± SD)	4.6 ± 1.9	13.2 ± 3.1
Mean number of laparoscopic procedures performed	85 ± 40	420 ± 110
Mean number of laparoscopic suturing cases	35 ± 20	260 ± 75
Primary specialty—general surgery, n (%)	10 (45.5%)	5 (45.5%)
Primary specialty—gynaecology, n (%)	7 (31.8%)	4 (36.4%)
Primary specialty—urology, n (%)	5 (22.7%)	2 (18.1%)

**Fig. 2:**
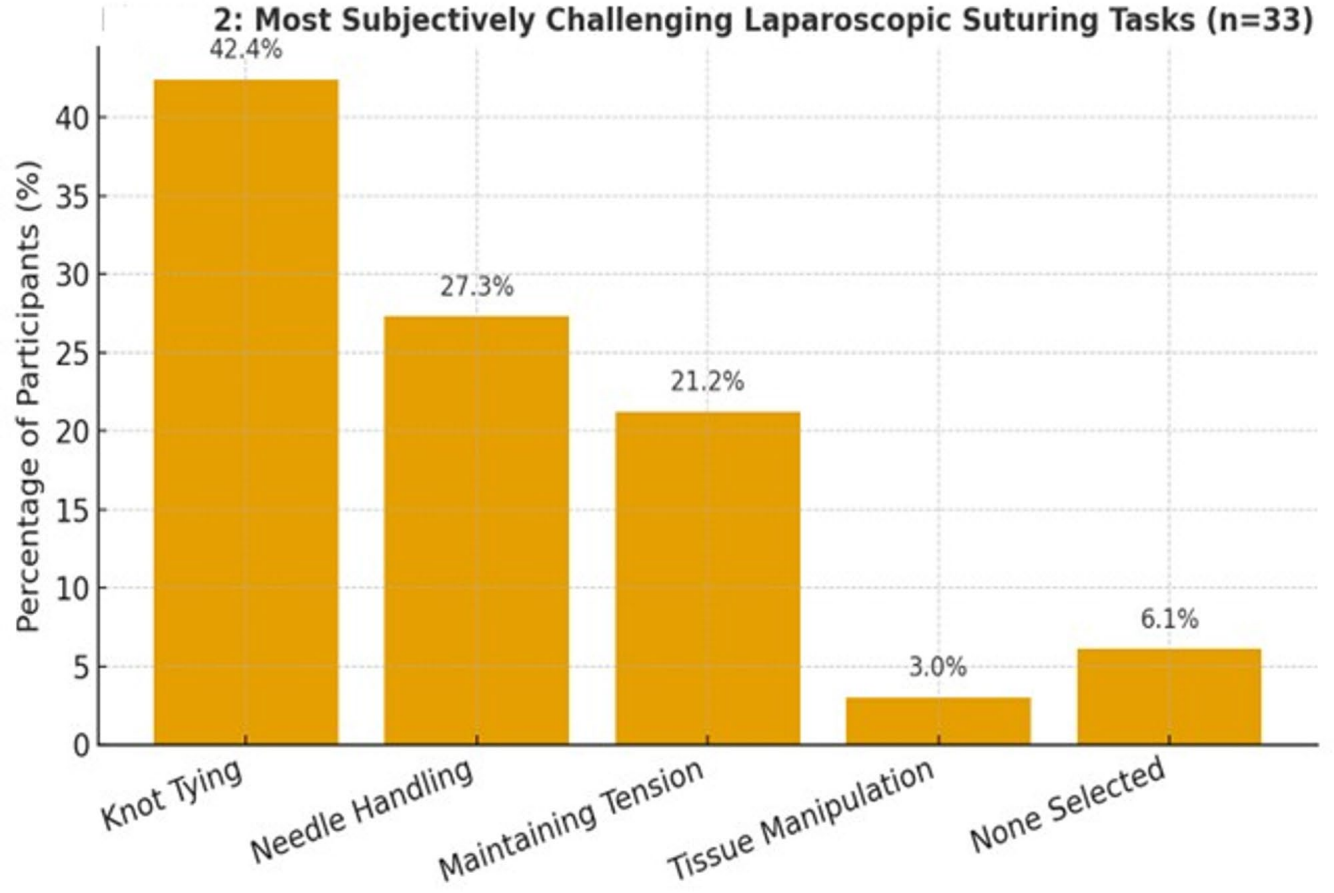
Showing the most subjectively challenging laparoscopic suturing tasks among all surgeons (novice + expert combined).

The distribution of responses regarding the most challenging aspects of laparoscopic suturing is summarized in Table 3; Fig. 2, there was no statistically significant difference in perceived difficulty in laparoscopic suturing between novice surgeons and expert surgeons.

**Table 3:** Distribution of subjective laparoscopic suturing challenges among novice and expert Surgeons.

Task	Novice surgeons (*n* = 22)	Expert surgeons (*n* = 11)	Total (*n* = 33)
Knot tying	11 (50.0%)	3 (27.3%)	14 (42.4%)
Needle handling	7 (31.8%)	2 (18.2%)	9 (27.3%)
Maintaining tension	4 (18.2%)	3 (27.3%)	7 (21.2%)
Tissue manipulation	0 (0.0%)	1 (9.1%)	1 (3.0%)
None selected	0 (0.0%)	2 (18.2%)	2 (6.1%)

**Fig. 3:**
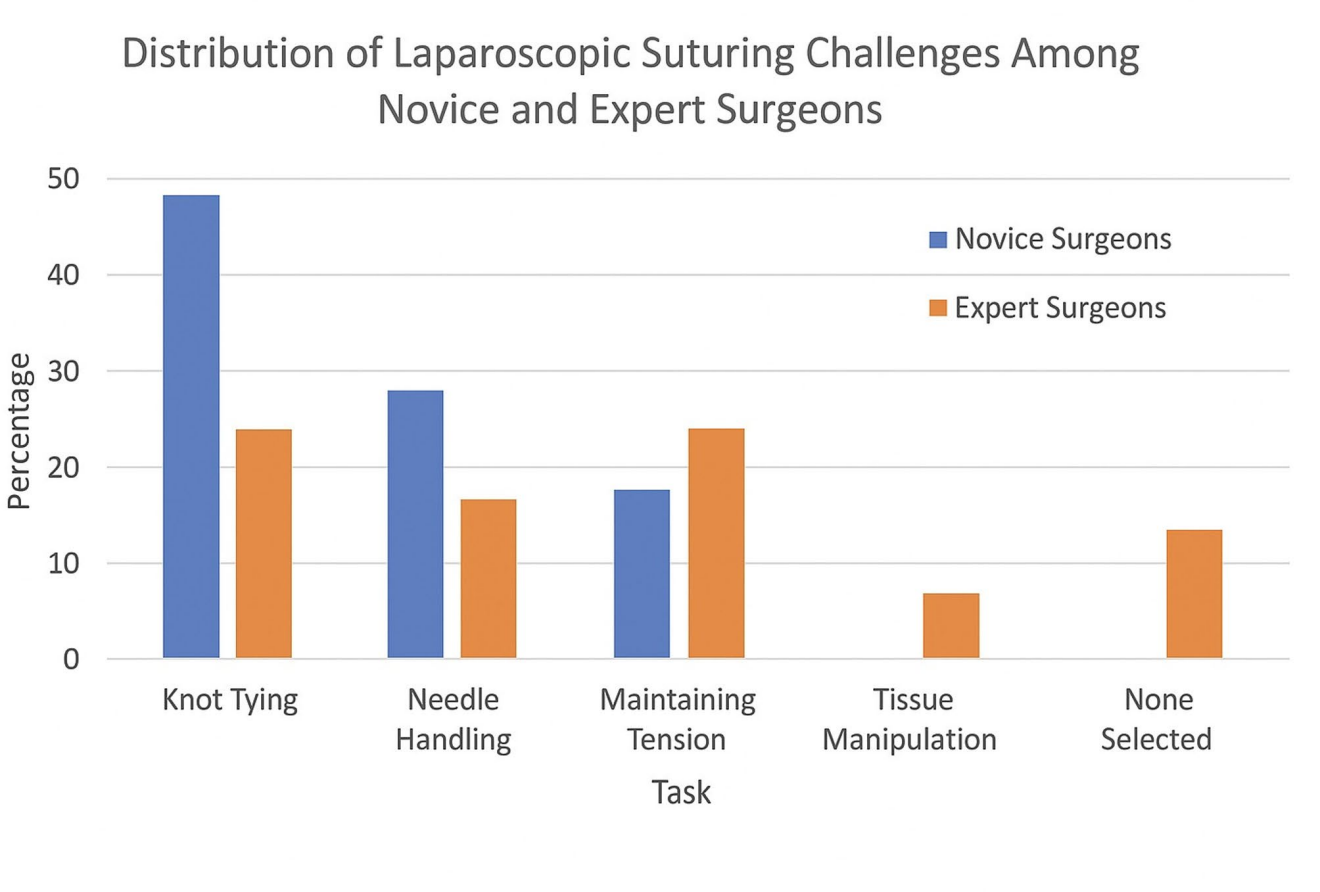
Bar chart comparing subjective challenges in laparoscopic suturing: novice versus expert surgeons.

Subjective analysis indicates that Knot tying was the most reported challenge, identified by 42.4% (*n* = 14) of all participants. Among novice surgeons, 50.0% (*n* = 11) reported knot tying as their primary difficulty, compared to 27.3% (*n* = 3) of expert surgeons. Needle handling was the second most frequently mentioned challenge, selected by 27.3% (*n* = 9) of participants from both novices and experts. This difficulty was more commonly reported by novice surgeons (31.8%, *n* = 7) than expert surgeons (18.2%, *n* = 2) as shown in Table 3; Figs. 2 and 3. Maintaining tension during suturing was noted as a challenge by 21.2% (*n* = 7) of participants, with a slightly higher proportion of expert surgeons (27.3%, *n* = 3) compared to novice surgeons (18.2%, *n* = 4) reporting this issue. Tissue manipulation was the least frequently reported challenge, with only one expert surgeon (9.1%) selecting it. Notably, two expert surgeons (18.2%) did not identify any specific challenge, while all novice surgeons selected at least one difficulty as shown in Table 3; Fig. 3. The “None Selected” category indicates that some surgeons, particularly two expert surgeons (18.2%) in this study, did not identify any of the listed tasks as difficult. This suggests that these surgeons felt confident in their laparoscopic suturing abilities and did not encounter significant challenges with the tasks presented. These participants likely possessed a high level of proficiency, leading them to perceive no specific task as particularly challenging.

**Fig. 4:**
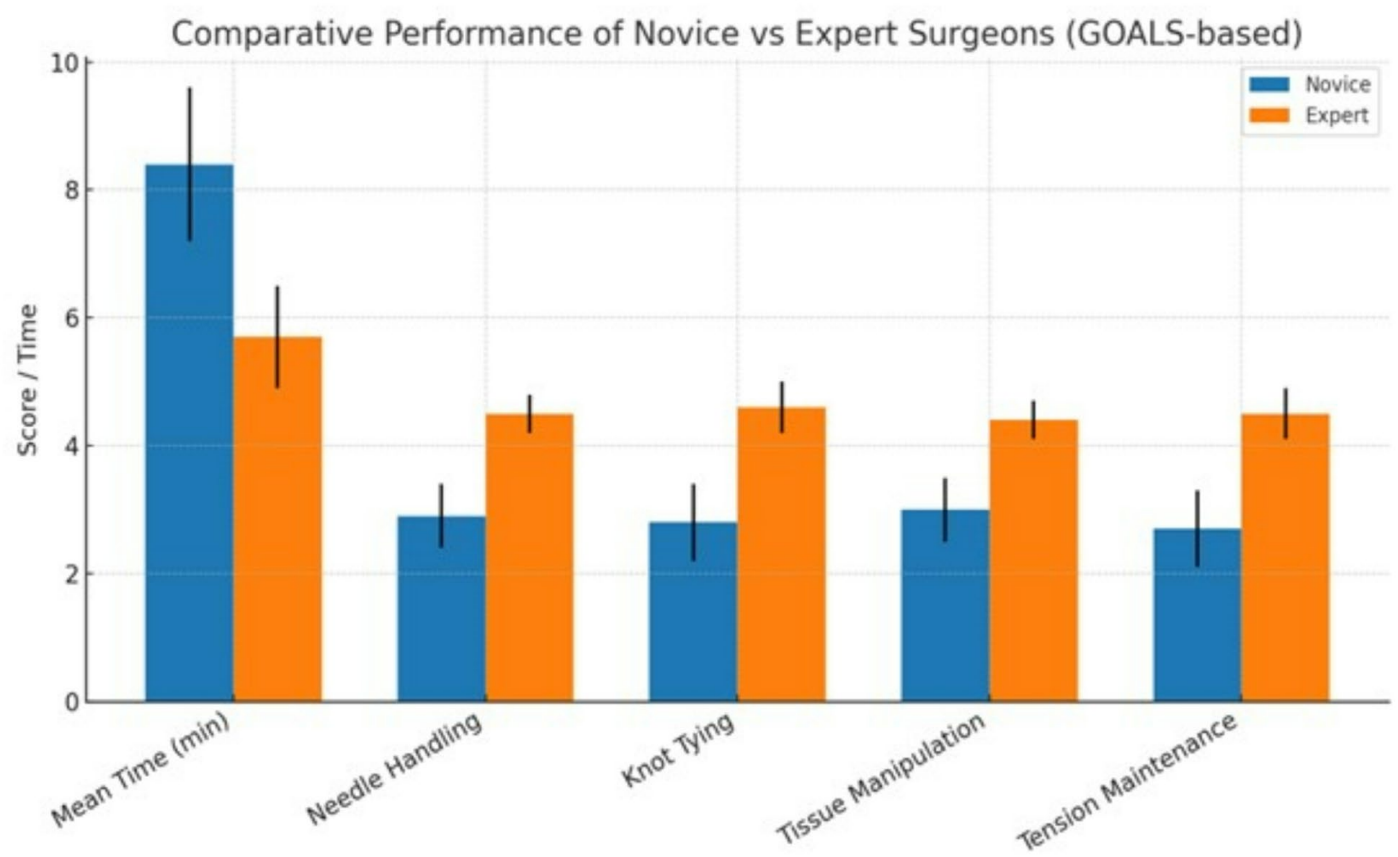
Comparative bar graph of objective performance metrics for novice and expert surgeons. Error bars indicate standard deviations. Performance parameters include time, needle handling, knot tying, tissue manipulation, and tension maintenance.

Objective video analysis was performed to compare the suturing performance of novice and expert surgeons. As shown in Table 4 and Fig. 4, expert surgeons completed suturing significantly faster than novices, with a mean time of 5.7 ± 0.8 min compared to 8.4 ± 1.2 min for novices (*P* < 0.001). Assessment using the GOALS scoring system demonstrated that experts consistently outperformed novices across all technical domains. Specifically, mean scores for needle handling were 4.5 ± 0.3 for experts versus 2.9 ± 0.5 for novices (*P* < 0.001), knot tying quality was 4.6 ± 0.4 versus 2.8 ± 0.6 (*P* < 0.001), tissue manipulation was 4.4 ± 0.3 versus 3.0 ± 0.5 (*P* < 0.001), and tension maintenance was 4.5 ± 0.4 versus 2.7 ± 0.6 (*P* < 0.001). These results indicate that expert surgeons exhibit superior speed, precision, and technical control during laparoscopic suturing compared with novices as shown in Table 4.

**Table 4:** Performance comparison between novice and expert surgeons using GOALS.

Parameter	Novice surgeons (*n* = 22)	Expert surgeons (*n* = 11)	*p* value
Mean time to complete suturing (min)	8.4 ± 1.2	5.7 ± 0.8	< 0.001
Needle handling (GOALS 1–5)	2.9 ± 0.5	4.5 ± 0.3	< 0.001
Knot tying quality (GOALS 1–5)	2.8 ± 0.6	4.6 ± 0.4	< 0.001
Tissue manipulation (GOALS 1–5)	3.0 ± 0.5	4.4 ± 0.3	< 0.001
Tension maintenance (GOALS 1–5)	2.7 ± 0.6	4.5 ± 0.4	< 0.001

All parameters except time are scored on the GOALS 1–5 scale. 1 = poor performance, 5 = excellent performance. Time remains an objective measure to capture efficiency. This table now fully reflects a GOALS-based evaluation, including needle handling, knot tying, tissue handling, and tension control.

Figure 4. Comparative bar graph of objective performance metrics for novice and expert surgeons. Blue bars represent novices, orange bars represent experts, and error bars indicate standard deviations. Performance parameters include time, needle handling, knot tying, tissue manipulation, and tension maintenance.

## Discussion

The findings of this study provide important insights into the persistent challenges faced by surgeons during laparoscopic suturing, particularly in knot tying and needle handling. Both novice and expert surgeons reported difficulties in these areas, suggesting that proficiency in laparoscopic suturing is not solely dependent on years of experience but also requires ongoing refinement through targeted training.

The inclusion of surgeons from multiple specialties reflects the multidisciplinary nature of contemporary laparoscopic practice and enhances the external validity of the findings, as summarised in Table 2. Although participants from general surgery, gynaecology, and urology were similarly distributed between novice and expert groups, differences in training pathways, procedural exposure, and learning environments may exist across specialties. The present study was not designed or powered to detect specialty-specific performance differences; however, this represents an important area for future research. Larger, specialty-stratified studies may help determine whether variations in clinical exposure or training environments influence the development and refinement of laparoscopic suturing skills. Subjective responses indicated that knot tying remained the most reported challenge, particularly among novices, with needle handling and suture tension also posing notable difficulties. A small subset of expert surgeons reported no specific challenges, which may reflect the effects of accumulated experience, enhanced motor control, and optimized ergonomic technique. These observations align with prior research demonstrating that fine motor skills, particularly knot tying and needle handling, continue to challenge surgeons across varying levels of experience^21–23^. Once foundational technical skills are mastered, experts appear to shift their focus toward efficiency, precision, and procedural flow rather than overcoming individual technical hurdles.

Objective performance data further support these interpretations. Experts consistently outperformed novices across all assessed parameters, demonstrating superior speed, accuracy, and control during laparoscopic suturing. The concordance between subjective perceptions and objective performance underscores the progressive nature of skill acquisition, in which tasks initially perceived as difficult become automated with experience, allowing surgeons to concentrate on refinement and efficiency.

The integration of both subjective and objective performance measures provided a more comprehensive understanding of laparoscopic suturing performance. Novices not only perceived greater difficulty but also showed quantifiable deficits in speed and accuracy. Experts, in contrast, displayed better motion control and knot integrity, reflecting the importance of advanced motor learning and muscle memory. Importantly, even among experts, knot tying and needle handling remained common sources of technical challenge, suggesting that these aspects represent enduring bottlenecks in laparoscopic skill mastery.

The unique contribution of this study lies in its dual-layered analysis, combining subjective perceptions with objective performance metrics to pinpoint persistent micro-level challenges in laparoscopic suturing, specifically knot tying, needle handling, and tension control across experience levels. This approach provides novel, data-driven insight into the fine motor and cognitive components of surgical skill development. Furthermore, this study emphasizes the role of ergonomic efficiency and cognitive control in performance quality. Informal videographic observations suggested that expert surgeons appeared calmer and more deliberate in their movements, while novices showed stress-induced hand adjustments and pauses, particularly when exerting higher suture tension prone to tissue tearing. These observations are anecdotal, as no formal motion or stress metrics were collected, and should be interpreted cautiously as an area for future research. This distinction highlights the potential role of cognitive resilience and ergonomic awareness in surgical proficiency.

One of the key distinctions of this study, compared to previous research on laparoscopic skills training, is its detailed examination of the specific technical challenges encountered during suturing, rather than offering a general assessment of overall surgical proficiency^7,10,24–28^. While previous studies have primarily focused on trends in skill acquisition and the implementation of structured training programs, this study provides a more comprehensive analysis^27,29–34^. By breaking down the intricate steps involved in knot tying and needle handling, this research offers a clearer understanding of the technical barriers that persist across all levels of experience. This micro-level perspective enables the identification of targeted interventions that can be integrated into surgical training programs to better address these challenges. Rather than simply reinforcing general laparoscopic skills, training can be designed to deconstruct complex suturing techniques into smaller, more manageable steps, facilitating more effective skill acquisition. These findings offer significant educational value by contributing to the enhancement of laparoscopic suturing training programs.

A key finding from this study is that novice surgeons struggle with several critical aspects of suturing that expert surgeons perform with greater ease, including precise thread handling, effective rotation of forceps for needle manipulation, proper positioning of the needle before grasping, and the ability to use the natural curvature of the needle for secure knot tying. These difficulties were consistently noted through post-hoc, unquantified observations, and future studies would be needed to formally measure their prevalence and impact. Most surgeons did not apply supination of the hand, which helps in forming a loop during knotting. Future simulation exercises could incorporate targeted modules focusing on these technical deficiencies; however, these recommendations should be considered observational hypotheses rather than definitive conclusions. Additional modules should emphasize correct wrist articulation, visual field alignment, and force modulation. One promising approach to addressing these challenges is the integration of advanced simulation exercises that focus on refining these specific movements. By incorporating step-by-step guidance and allowing for repetitive practice of these essential skills, trainees may develop greater dexterity and confidence in performing laparoscopic suturing. Additionally, this study explored cognitive and psychological dimensions of laparoscopic performance, particularly the “mentality” of the operator. Informal videographic observations indicated that expert surgeons appeared calmer and more deliberate in their movements, while novice surgeons often exhibited visible signs of stress, frequent pauses, and reactive hand adjustments with rushed movements; these behaviours were not formally measured. Recognizing these patterns may help guide the development of training programs aimed at enhancing not only technical skill but also cognitive resilience and the ability to maintain focus under pressure.

Another key distinction of this study is its detailed qualitative examination of the technical challenges encountered during suturing. Informal videographic observations of hand motion, wrist rotation, and needle positioning during tasks suggested that ergonomic efficiency and deliberate instrument control may contribute to improved knot quality and task accuracy, although these aspects were not formally measured. The quantitative differences between groups, particularly in time efficiency and tension control, support this observation, indicating that refined ergonomic awareness likely translates to measurable improvements in performance metrics. Collectively, these findings suggest that combining cognitive awareness with focused technical training may provide a more comprehensive understanding of laparoscopic skill development and performance improvement.

Furthermore, the data generated from this study could serve as a foundation for developing artificial intelligence (AI) and machine learning (ML) systems designed to master laparoscopic suturing skills, objectively assess performance, and shorten the proficiency curve for this operative skill^35,36^. Similar AI-based systems have already been successfully applied to other procedures, such as laparoscopic cholecystectomy^37–39^. The use of AI-based feedback could play a transformative role in addressing these challenges^40–42^. AI-driven training platforms have the potential to provide real-time, personalized feedback by analysing hand movements, force application, and instrument positioning^43^. These systems can detect errors and suggest corrective actions, allowing trainees to refine their techniques more effectively. The integration of AI in surgical training could bridge the gap between novice and expert-level proficiency by providing an adaptive learning environment that customizes feedback based on individual performance metrics^44^.

While this study offers valuable insights into the challenges associated with laparoscopic suturing, several limitations should be acknowledged. The relatively small sample size, although diverse in terms of experience levels, may limit the broader applicability of the findings. Future studies should include larger cohorts of surgeons from multiple institutions and specialties to provide a more comprehensive understanding of laparoscopic suturing difficulties. To address potential biases introduced by self-reported data, future research should integrate objective data collection tools such as instrument tracking systems and performance scoring rubrics, enabling cross-validation of subjective perceptions against empirical performance. The reliance on self-reported difficulty ratings introduces an element of subjectivity, influenced by personal learning curves, prior experience, and internal benchmarks. Additionally, the informal nature of observed motion differences and stress-related behaviours highlights the need for formal measurement in future studies. The use of mobile applications or AI-based software capable of analysing hand movement smoothness, instrument trajectory, and motion efficiency could enhance the precision of performance assessment. By combining subjective self-assessments, objective performance data, and app-based motion analytics, future research could achieve a more accurate and comprehensive understanding of laparoscopic skill acquisition and performance improvement.

## Conclusion

This study underscores that technical challenges in laparoscopic suturing, particularly knot tying, needle handling, and tension control, persist across all levels of surgical experience, highlighting the need for targeted and specialized training strategies. This research offers a comprehensive, multi-dimensional assessment of suturing proficiency by integrating subjective questionnaire responses with objective video-based scoring including parameters such as time, needle handling, knot quality, tissue manipulation, and tension maintenance. Rather than emphasizing general laparoscopic skills, training programs could benefit from focused simulation exercises that deconstruct complex suturing tasks into discrete, manageable components, enabling repeated practice of critical micro-skills such as loop formation, wrist rotation, and force modulation.

## Data Availability

The original contributions presented in this study are included in the article/supplementary material. Further inquiries can be directed to the corresponding author(s).
